# Health Assets, Vocation and Zest for Healthcare Work. A Salutogenic Approach to Active Coping among Certified Nursing Assistant Students

**DOI:** 10.3390/ijerph17103586

**Published:** 2020-05-20

**Authors:** Natura Colomer-Pérez, Elena Chover-Sierra, Vicente Gea-Caballero, Joan J. Paredes-Carbonell

**Affiliations:** 1 Department of Nursing, University of Valencia, 46010 Valencia, Spain; elena.chover@uv.es; 2 Development and Advising in Traffic Safety (DATS) Research Group, INTRAS (Instituto de Investigación en Tráfico y Seguridad Vial), 46022 Valencia, Spain; 3 Consorcio Hospital General Universitario de Valencia, Medicina Interna, 46014 Valencia, Spain; 4 Escuela de Enfermería La Fe, Centro adscrito Universitat de València, 46026 València, Spain; vicentegeacaballero@gmail.com; 5 Grupo de Investigación GREIACC, Instituto de Investigación Sanitaria IIS La Fe, 46026 València, Spain; 6 Centre de Salut Pública d’Alzira, Conselleria de Sanitat Universal i Salut Pública, Alzira, 46600 València, Spain; paredes_joa@gva.es; 7 Fundació per al Foment de la Investigació Sanitària i Biomèdica de la Comunitat Valenciana (FISABIO), 46035 València, Spain

**Keywords:** salutogenic model of health, health assets model, asset-based approach, nurse, certified nurse assistant, vocation, active coping

## Abstract

People’s health assets (HA) mapping process and design dynamization strategies for it are paramount issues for health promotion. These strategies improve the health heritage of individuals and communities as both the salutogenic model of health (SMH) and health assets model (HAM) defend. Connecting and mobilizing HA and strengthens the ‘sense of coherence’ (SOC) are both related to enhancing stress active and effective coping strategies. This study aims to describe the HA present in a population of certified nursing assistant students (*n* = 921) in Spain and then to explore their relationships with the SOC, the motivation to choose healthcare studies and their academic performance. A great variety of HA were identified and mapped. Findings showed that individuals with greater motivation towards self-care and ‘caring for others’ as internal HA, possessed higher SOC levels and a strong vocation for healthcare work. Differences in HA were identified according to gender, age and employment situation. Consistent connections between the care–relation factor and vocational factor with interpersonal and extrapersonal HA were reported. Evidence and results substantiated the salutogenic and asset-based approach as a proper strategy to strengthen SOC, dynamize their HA map, reinforce the *sense of calling and* enable Certified Nurse Assistant (CNA) students to buffer against caregiving-related stress and thrive in their profession.

## 1. Introduction

### 1.1. Salutogenesis and the Health Asset Framework as Models to Cope with Stress

Even though the formal Salutogenic Model of Health (SMH) has not received enough attention in research and practice since its origins, there is a renewed global interest in seeking for future directions for the concept. It emerges from the need for a better understanding of the theory and its implications addressing the full spectrum of the human health experience [1]. Likewise, the concept of health assets is becoming increasingly popular and it was explored in different settings and populations throughout the world [2]. Thus, health promotion from a positive health paradigm is grounded in two frameworks: Aaron Antonovsky’s salutogenic theory and the Asset–based community development (ABCD) approach [3,4]. The identification of people’s health resources and assets and the design of dynamization strategies for them is a paramount issue by improving the health heritage of individuals and communities as it is defended by the SMH and the Health Assets Model (HAM) [4,5,6,7,8].

In this sense, the salutogenic approach promotes the concept that when people can make sense of the world that surrounds them, they will also notice a correspondence between their actions and the effects these actions will have on their environment [9,10]. In this regard, there are closer connections between salutogenesis and a health asset-based model. The salutogenic assumption seeks to explore the origin and stability of health by understanding how it can be created and determines the optimum conditions for its development [11,12]. On the other side, the HAM establishes that: the more possibilities someone has to experience and accumulate positive effects of a series of assets throughout their life, the higher the chances of achieving health goals are [4]. To this effect, HAM implements community intervention’s methodologies (such as social participation and action research) to develop a salutogenic health promotion strategy. This strategy is usually addressed in four phases: identification of protective factors, participatory asset mapping, connection and dynamization of health assets (HA) and finally, evaluation [13,14].

In parallel, those approaches also maintain interesting and close linkages to ‘Stress and Coping’ Lazarus and Folkman’s theory. These authors address the existence of internal, interpersonal (conceived as social support) and external factors to individuals that buffer adverse effects of stress, allowing people to develop mechanisms to regulate emotional responses to stressful circumstances and having a high impact on well-being. Then, stress is a two-way process that involves stressors produced by the environment and the individual subjective responses to them by using primary and secondary cognitive appraisals [15,16]. Hence, these factors may be identified, equated and assimilated on many occasions to the salutogenic ‘general resistant resources’ (GRR) defined by Antonovsky [9,10] because they also include psychological traits, coping strategies, social and cultural factors and social support. Moreover, these factors contribute to increasing people’s resilience, enabling them to solve problems adaptively, assessing stressful events as meaningful, predictable and manageable [17]. All in all, the theory posits that life experiences shape the sense of coherence (SOC)—the core element of the SMH—that helps to mobilize resources to cope with stressors and manage tension successfully [18].

In exploiting the perspective’s whole meaning, the present research assumes the notion of these resistant resources consolidated as health assets. Consequently, to operationalize all these factors for health-promoting purposes, both the SMH and HAM advocate for categorizing the intrapersonal, interpersonal and extrapersonal health elements which operate as protective and promoting factors to buffer against life’s stressors [19]. A health asset itself can be defined as any factor (or resource) which enhances the ability of individuals, groups, communities, populations, social systems and institutions to maintain and sustain health and well-being and to help to reduce health inequities. These assets can operate at the level of the individual (for example, abilities, competences and talents), group and community (including the role of supportive networks and population as protective or promoting factors to buffer against life’s stresses) and eventually an organizational or institutional level (for example making use of external financial, physical or even environmental resources) [19]. According to the management of stress, literature advocates that all those resources not only immediately help people to cope better with stress and surviving [1]; but also, over time, personal and environmental resources can help with recovery and healing [20,21,22,23], even from early life adversities in adult populations [24].

### 1.2. Salutogenic Active Coping and Zest for Work in Healthcare Professionals

Advancing and empowering the SMH and HAM to understand better the ways of coping productively with stress seems to be a paramount purpose. Furthermore, this challenge must be primarily tackled in health care professionals and their prior academic and formative context. Regarding the case of CNA nursing students and their future job demands envisaged, stress is a psychosocial factor that influences the academic performance and well-being of this group [25]. Nursing students not only face academic, but also face pressure at work during their training period [26]. Like previous findings, care behaviors correlated negatively with depression, distress and emotional exhaustion and positively correlated with coping strategies and a positive attitude to one’s role at work [27]. Not surprisingly, the negative consequences of not having adequate coping strategies to undertake the inherent demands of nursing degree, as well as the future professional life, have an impact on their health and mental well-being. Furthermore, this situation is also directly related to professional performance [28].

Overall, it was shown that those using a greater variety of health assets can develop a greater sense of coherence (SOC) that will also allow them to promote active and effective stress coping strategies [18]. Concretely, healthcare students and workers with strong SOC may perceive and appraise the demands of their work environment as challenging rather than threatening, according to Antonovsky’s research on health-promoting factors at work [9]. In addition, active coping is a valuable asset, especially in very demanding situations that nurses have to face up every day; therefore, resilient professionals are vital to the proper functioning of a health system [29]. Given those facts, researchers and professors suggest that daring to strengthen and reinforce the salutogenic capacity of the students must be expanded as part of the professional training in healthcare professional’s degrees in order to promote and maintain the engagement and the zest for healthcare work [30]. More recently, it was observed the impact of the motivational factor in job engagement mediated by a sense of calling. This calling–vocation match brings forth from introspection, sensibility and reflection, which produces a working situation that for the most part, feels deeply gratifying and meaningful to the individual, resulting in zest for work and vitality [31].

In the spirit of the whole latest reflections on the salutogenic paradigm, for a better conceptualization of salutogenic orientation, it is necessary to encourage alternative approaches, including qualitative research [1].

Conversely, the health asset literature is underdeveloped, and its sustained credibility depends on future research dealing with definitional, theoretical and evaluative issues, being, therefore, imperative accomplishing more research to deeply apprehend the health assets model in a global context [2]. Thus, the pursuit of a better understanding between the salutogenic perspective (measuring SOC) and a health–asset approach (observing reported health assets) is the primary purpose of this study, to tempt a potential and early incorporation of a salutogenic orientation in healthcare-providers’ studies. To this effect, the first phase of the present study has explored the salutogenic paradigm among nursing assistant (CNA) students in a region of Spain. Based on those findings, it seems that possessing a strong SOC appears to contribute towards improved resistance to stress, which in part, may also justify the motivation for studying a career that is pleasing and obtaining high academic performance despite being a profession with high demands and marked stressors [32]. Additional analyses of this research also have confirmed that CNA students referring a good practice on self-care and the willingness to caring for others (described as an internal health asset) also display an optimal zest for work in the nursing discipline [33].

This current study faces the last phase of the research seeking the opinions of CNA students about the HA that provides opportunities for well-being and health and determines a Health–asset Map articulated by participants using mixed-methods. Subsequently, it intends to explore, thorough a quantitative approach, the relationships between those HA, the SOC, the sense of calling (vocation-motivation variable) to choose healthcare studies as a career in concert with the academic performance for this certification in public education and vocational centers (Comunitat Valenciana, Spain).

## 2. Materials and Methods

### 2.1. Study Design and Sample

Mix-method study (qualitative and quantitative study: cross-sectional, analytical and exploratory) was carried out in 2016. Participants were enrolled—at data collection time-, in the last semester of certification of nursing assistant (CNA) from the total of public upper secondary schools providing vocational education and training (VET) certifications at Comunitat Valenciana (Spain). The study was aimed at the entire student population (*n* = 1150) enrolled in the region. With an IC = 95% and an error = 5%, a minimum sample of *n* = 289 was required.

### 2.2. Data Collection

Sociodemographic data collected were: (a) gender (male, female), (b) age (categorized: <30, 30–45, >45), (c) employment status (employed, unemployed), (d) income level (net income of the student’s household, understood as the level of income received, from among the following options: high, medium/high, medium, medium/low, low, does not know/does not answer), (e) public secondary education centers in which CNA studies are taught, (f) geographical emplacement of the center (rural, urban, large city), (g) self-reported academic performance: students were asked about their academic record at the end of the last semester—when they already knew their global marks- and the responses were: fail (<5), pass (5–5.9), good (6–6.9), remarkable (7–7.9), outstanding (8–8.9), with distinction (9–10); in Spain, the academic record is scored in a scale of 0–10, with 10 being the highest score to reach and below 5 is considered as failed), (h) motivation of choice of studies (vocational, could not be enrolled in other studies, seek for better employment option, unmotivated). Some opened questions served to identify HA (intrapersonal, interpersonal, extrapersonal), defined as things/people/places that increased their well-being. SOC levels (a global orientation of the personality that facilitates the solution of problems in an adaptive way in stressful situations to which people are subjected throughout their lives) were assessed by the orientation-to-life questionnaire—13 items (OLQ-13 or SOC-13) [34]. This 13-item questionnaire also measures the dimensions of *comprehensibility* (with 5 items), *manageability* (with 4 items) and *meaningfulness* (with 4 items). The SOC-13 scale has shown good internal consistency, with a Cronbach’s alpha between 0.70 and 0.92 [34,35,36].

### 2.3. Procedure

Professors from all educative centers attending Nursing Assistant public certifications were first contacted to mail them the questionnaire. Students completed a self-administered online questionnaire (with internet protocol—IP-response restriction) during their schedule’s classes collecting qualitative data: HA; and quantitative data: the sense of coherence scale (SOC), factors related (motivation to study this career and self-reported academic performance) and sociodemographic variables. The questionnaire included information about the study and the contact details of the principal investigator. There were no exclusion criteria, and permission to participate in the study and consent to use the data were required. The qualitative analysis was carried out to identify the different types of HA, categorizing them into the categories already proposed. Subsequently, additional quantitative analysis was also carried out.

### 2.4. Data Analysis

#### 2.4.1. Qualitative Phase

The CNA student’s HA mapping was underpinned according to the fundamentals of HAM methodology [3,13,37,38,39]. Most of these authors propose six categories of health assets: people, agencies or organizations (with or without profit), institutions, infrastructure or physical resources, economy and culture (including traditions, identity and sense of belonging). In this study, HA were collected and categorized in four HA groups, according to recommendations and results of previous research in this field [40]. First, the intrapersonal HA, which corresponds to an individual level; second, the interpersonal HA; third, the extrapersonal HA I—as institutions, organizations, etc.-; and finally, the extrapersonal HA II—as infrastructures, indoor/outdoor spaces, etc.-, which correspond to a community level.

In order to dump qualitative data collected from the questionnaire regarding to HA, the procedure was developed in 3 operationalization’s phases and conducted by the main researcher and a different extra researcher. Phase (1) first, consisted of an information’s transcription of the given responses from the open-ended asset questions, through a thematic analysis. This analysis was employed to codify information, also using the word economy, making significant groupings of the answers whenever possible, and trying to preserve the literalness of the discourse. Phase (2) was a reflection stage to prepare emerging subcategories for the four HA types that included sensitizing concepts. This means that these subcategories were aroused as a result of raising significant reference frames from the thematic analysis. Thus, a total of 30 HA’s subcategories were built and identified with a subsequent numeric code: 3 subcategories for the Intrapersonal HA, 7 subcategories for the Interpersonal HA, 8 subcategories for the Extrapersonal HA I and 12 subcategories for the Extrapersonal HA II. Phase (3) was a proceeding of classifying and reconversion of each thematic content in its related HA subcategory—concretely, into its code number- with the purpose to prepare the database for the posterior statistical analysis.

In parallel and once again following the HAM methodology, the graphical students’ HA map was built as a reflection of the literal qualitative data collected at the open HA questionnaire.

#### 2.4.2. Quantitative Phase

Both the population characteristics and the subcategories of the HA classification that emerged after the qualitative analysis were analyzed in this phase. Descriptive statistics were applied to obtain frequencies and percentages in case of qualitative variables or means (M) and standard deviations (SD) to describe the quantitative ones.

chi-squared test was used to analyze the relationship among the HA identified and some population characteristics. Differences in SOC scores (global and for each dimension), according to the HA subcategory, were analyzed using the nonparametric Kruskal–Wallis test. In case of finding differences in SOC scores among groups, post hoc analysis using Bonferroni correction was performed to identify between which groups these differences occur.

In all cases, statistical significance was set at *p-*value < 0.05.

### 2.5. Ethical Considerations

In the case of underage students, prior authorization was obtained from parents or legal guardians to participate in the study. At the time the questionnaire was administered, the following measures were taken to ensure the anonymity and protection of the study participants: the professors—who were instructed to give the relevant indications to answer questionnaire right and accurately informed students that their participation was voluntary. They were also informed that not participating in the study did not imply grievances for them. The first screen of the online questionnaire informed about the legal details of the research. Data were anonymized and processed according to the recommendations of the State Data Protection Agency based on Organic Law 15/1999 and the European Directive on Data Protection 95/46/EC. Furthermore, permissions were also requested and obtained from each educational center and the competent organism in the area of education in the region (05ED01Z/2016/406/S) Resolution of February 25th, 2016 of the Autonomous Secretariat of Education and Research of the Conselleria d’Educació, Investigació, Cultura i Esport.

## 3. Results

### 3.1. Characteristics of the Participating Students

921 students answered the questionnaire voluntarily, 751 were women (81.54%) and 170 were men (18.46%). This number of responses meant 80.09% of the population of CNA in the Valencian Community. The average age of the participants was 28.52 (SD = 11.43, Range = 16–57).

The characteristics of the studied population studied are shown in Table 1.

### 3.2. Qualitative Analysis: Mapping the HA Identified by CNA Students

**Figure 1:**
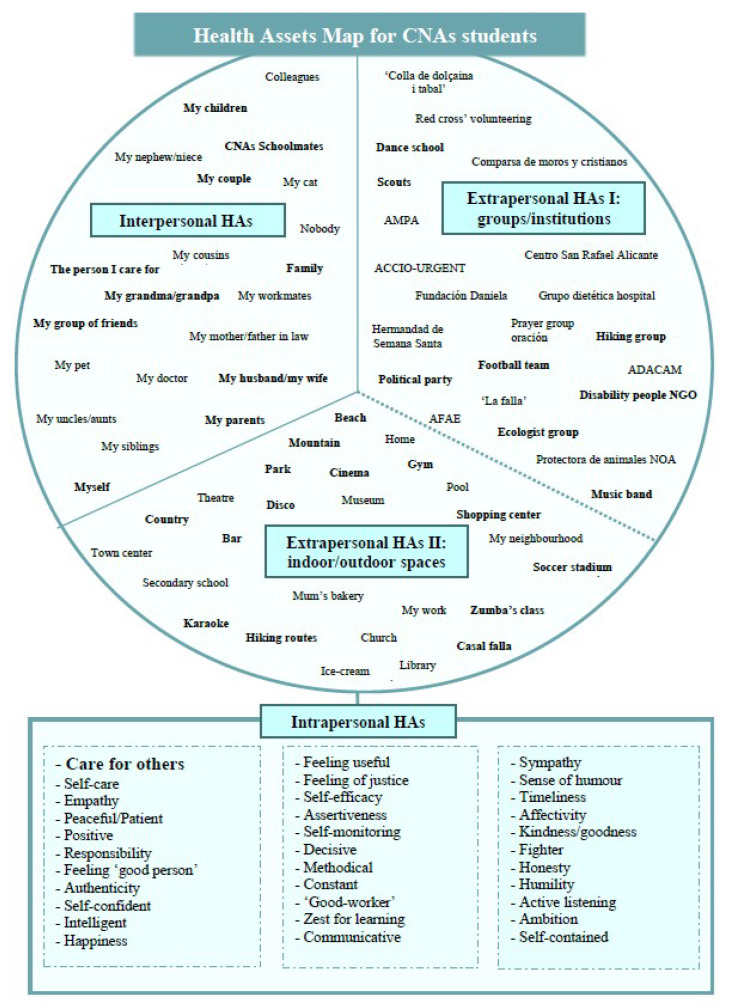
Map of health assets (HA) articulated by CNA students.

The qualitative analysis of the answers offered by the participants allowed the researchers to design a qualitative map of HA identified by the CNA students, which is shown in Figure 1.

### 3.3. Quantitative Analysis of HA Identified by CNA Students

The quantitative analysis of the students’ responses is presented in Table 2, which shows the frequencies of each one of the different HA identified by the participants.

According to HA identified in this study, it is observed that in the case of intrapersonal ones, a great variety of them was identified by the CNA students. However, 9% of students refer to the fact of “caring for others” as one of the prior inner assets. Regarding interpersonal HA, the couple and the family nucleus (more or less extensive) were identified by up to 89% of students and the group of close friends by 6.4% of them. Finally, and concerning extrapersonal HA, a high number of groups and institutions were identified. On one hand, it is essential to highlight the social group (friends, classmates)—which was identified as an asset by 43% of students—and sporting institutions, identified by 15.4% of students. In addition, a wide variety of physic spaces was also identified by these students; however, they highlighted the recreational spaces (26%) and natural outdoor spaces (beach by 14.2% and mountain by 13.6%) and or specific spaces to do sports activities (6.8%). Interestingly, another 11.5% also identified their own home as an asset for well-being and health.

When analyzing the HA identified by the participants based on specific descriptive characteristics of the population, several differences are found, some of them statistically significant (chi-squared test). Table 3 shows these differences according to the gender, age and employment situation of CNA students.

### 3.4. Relationship between SOC and HA

The results on the SOC-13 obtained by this population and its relationship with academic performance can be read in a previously published study [32]. However, a summary is exposed below: the mean score (M) for total SOC measurement was 56.38 (SD = 12.24). Regarding the SOC dimensions, the average score for each subscale was: (i) *manageability* Mean = 16.45 (SD = 4.53); (ii) *comprehensibility* Mean = 19.27 (SD = 5.642; 30) and (iii) *meaningfulness*, Mean = 20.65 (SD = 4.48; 23).

When studying the relationship between the intrapersonal HA identified and the scores in SOC, it is found that those students who outlined aspects related to ‘taking care of others’ got higher scores in SOC than those who identified other introspective features of behavior, although these differences were not statistically significant (Kruskal–Wallis test). All these differences are shown in Table 4.

In the case of the relationship with interpersonal HA, shown in Table 5, it is found higher values of SOC in those who identified their children as health and well-being generating factors than those who identified other members of their family or their group of friends. These differences were statistically significant (Kruskal–Wallis test).

The post hoc study to analyze intergroup differences found in some cases statistically significant differences (*p* < 0.007, with Bonferroni correction) in SOC scores obtained by students who identified their descendant relatives (the highest values) as HA and those who identified the other interpersonal HA. Table 6 shows mean differences in SOC scores, when comparing them among groups, considering as a reference those who identified their descendent relatives as HA.

In this post hoc analysis, other statistically significant differences were found when comparing scores obtained in other groups such as in the case of people who identified as HA their parents and people who identified their family and friends as HA, but only in the scores of SOC *meaningfulness* dimension (*p* = 0.003).

Eventually, concerning extrapersonal HA I (groups or spaces), as shown in Table 7, individuals that referred to using open sporting spaces as a HA stood out for higher scores in SOC. In contrast, those who referred to cultural spaces (theatres, cinemas, museums, etc.) showed an average of around 6 points less in SOC. Higher values of SOC with students who used to accomplish volunteer actions and those who identified educational institutions as a health and well-being asset were also perceived. These differences were not statistically significant in all cases (Kruskal–Wallis test).

Post hoc analysis to study differences among CNA students according to extrapersonal HA I did not show statistically significant differences in SOC global scores. Nevertheless, in the case of the comprehensibility dimension, students who considered religious institutions as a HA got the lowest scores; these significant differences were found when comparing their scores with those achieved by the students who identified other extrapersonal HA I, such as sporting or educational institutions or their social group (*p* < 0.006, with Bonferroni correction). Furthermore, when comparing SOC global scores, according to HA II identified, no statistically significant differences were found.

### 3.5. HA According to Motivation for Choosing Nursing Studies

In light of exploring the calling for CNA’s work in our population, 48.2% of students showed a vocational orientation when choosing this career, which was also significantly related to their better self-reported academic records. Furthermore, they scored higher in SOC (both, globally and three dimensions separately), as published previously [32] and those were also the ones who most frequently identified the concept *care for others* within intrapersonal HA (85.7%).

When analyzing the interpersonal HA referred according to this motivational variable, it is observed that the students with a vocational orientation identified their own families as a prior factor providing well-being to them. As for both categories of extrapersonal HA prioritized by these vocation-motivated students, the most valued were to be optimally included in a social group (44.4%), natural spaces (27.7%) and recreational/leisure spaces (24.3%). A lower priority appears in cultural (1.8%) and religious institutions (0.7%).

## 4. Discussion

The purpose of this research was to describe the HA identified by a sample of CNA students and to establish a relationship with essential resources to deal with their learning (and life) environment. It was also interesting to explore if having a coherent life meaning—and the presence of attaining their personal goals through an academic achievement- showed relations with vocational factors like taking care of others or owning a *sense of calling* for nursing studies, as the results confirmed.

Concerning intrapersonal HA related to relevant values as ‘caring for others,’ this study has connected the willingness for care asset-value with consistent scores of SOC, especially were women who referred the chance to ‘taking care of others’ as an internal resource generating well-being. This aspect and other ones related to patience and fondness provided in care were also referred to as protective assets by informal caregivers of Alzheimer’s patients in a study developed by Agulló-Cantos [41]. Another study, which mapped internal HA thorough an intervention with resilient practices (mindfulness) in also informal caregivers determined that this technique—even it cannot be used by itself- could help them to manage a multitude of stressful situations and guarantee a good level of care for another person and even maintain their health and well-being [42]. Reviewing literature in educational contexts, a study mapping HA explained better academic achievement as a factor of well-being for students in a regular schooling experience [5], which justifies the importance of attending self-efficacy from a salutogenic perspective as the present study has observed. Linking to the essence of the salutogenic framework, several works have examined the relationships between SOC and school-related stress [43,44,45]. In the same line of this research, SOC correlated significantly and positively with school marks, school performance, achievement and success [32,46]. The present study determines that students relating this ‘caring for others’ asset-value also scored higher in SOC and those variables were significant related to students with a powerful sense of calling as a reason to perform this career. These findings reflect the proposal of Vinje [47] matching vocational element, giving meaningfulness to the chosen profession along with assuming and integrating healthcare strategies as combined, synergic and protective factors in health for nurses. This author even suggests that this chain of phenomena acts like a real snowball providing enthusiasm for the profession called zest for healthcare work, which explains the commitment to the practice of nursing and genuine job engagement [29,30].

In terms of highlighting the interpersonal HA pointed by CNA students, the ubiquity of positive social values provided by family and social network is consistent with findings from other studies which have found positive identity and positive social values [17] and the protective effects of supportive family relationships [48,49], as resources to cope with stressful life events. Concretely, women with children in the moment of the data collection were those who identified them as the main interpersonal assets in their lives. In contrast, men students referred to friendship as their mostly identified HA.

Observing the availability of social support resources in adolescents, they stressed the importance of family, friends and neighbors and the feeling of being supported and taken care of by their parents, community and friends constituting an essential social resource that contributed to resilience [50,51,52]. Regarding the school context, support from teachers and support from classmates seem to be critical elements during adolescence [6,53]. Besides that, stronger SOC scores were reported by students identifying their children as an interpersonal HA, as the post hoc results also confirmed, these students also obtained higher scores than individuals who identified another interpersonal HA. Based on the findings from the literature review, also those who identified other relatives and friends as HA scored slightly lower in SOC. These findings concur with a study conducted in Spain by Malagón [54], that reported higher SOC levels for nurses accounting for the satisfactory and supportive nuclear familiar network.

Finally, and discussing the extrapersonal HA, it was observed more frequency of response concerning using sporting institutions among CNA male students. In contrast, women identified preferences for educational institutions as healthy resources. These data lead to a reflection about the expression of a society that tends to perpetuate the assignment of roles and stereotypes to women and men in a differentiated way and the need to work in breaking these stereotypes from a salutogenic educational environment. Another interesting relation was observed between higher SOC scores and the fact to undertake some volunteer activities and referred to the frequent use of educational and cultural institutions. On the other hand, those that frequented religious institutions as a coping strategy scored quite lower in SOC at the time of data collection. Even though, in this case, the post hoc analysis did not find statistically significant differences. All in all, for this typology of assets, the results are consistent with those identified as HA in previous researches: neighborhood and community network, sociocultural environment and heritage, sporting and leisure time spaces and natural environments as the most relevant among the population [3,4,40,55,56].

At this stage and analyzing the specific HA map articulated by Valencian CNA students, some aspects must be tackled, such as the consistent connections of the care–relation factor and vocational factor with real health assets reported. Therefore, is the salutogenic and asset-based approach a facilitating strategy to allow CNA nursing students to strengthen SOC, reinforce their *sense of calling*, delve into the *zest for healthcare work* and consequently, enable them to buffer against work-related caregiving stress and thrive in their professions? Even more, could this holistic and positive health-promoting paradigm be early conducted at nursing VET schools and university nursing schools? The findings sustain partial aspects of this postulate. One research focused on undergraduate nursing education emphasized the importance of the role of some personal HA as self-efficacy, emotional intelligence and develop nursing professionalism as inherent aspects to be included in educational strategies for these healthcare students [57]. Mayer and Boness [58] suggested that educational contexts can play a crucial role in creating consistency and a safe and respectful learning environment that promotes social support and enhances SOC. In this regard, a systematic review from the UK released that an Asset–based community development proved a useful ‘lens’ to view research in schools on the interaction of education and health improvement, having confirmed that there are promising areas for health gain from using schools as ‘health assets’ [59]. Lindström & Eriksson [12] reflected on the importance of introducing the salutogenic framework in educational science by starting a discussion about the content of health education and health literacy expanding towards healthy learning, with emphasis on positive health promotion.

Then, the commitment seems to point towards the convenience for a decisive introduction of salutogenic orientation in nursing curricula. However, this academic engagement should probably be extended to the rest of health sciences’ disciplines as a core component of the study plans [57]. In addition, asset-based interventions must also be implemented in educative programs (as well as in continuing education) and the challenge goes through advocating for teachers being specially trained in salutogenic approaches. That is worthwhile because the salutogenic framework seems to provide a better understanding of the ways to tackle workload and cope with professional stressors, promotes positive health and well-being among future caregivers professionals and could improve, all in all, their efficacy as healing agents.

The implications of our study go in that direction. On one hand, it contributes to the theoretical development of the Salutogenic Model of Health and the Health Assets Model; both have shown to be adequate constructs to improve the health and well-being of any individual from a positive perspective, also in students [2,10]. Furthermore, this is relevant to the extent that this study contributes to reinforcing the growing existing evidence [1,4,6,13], based on non-theoretical population studies. According to the transference to a practical level, it is proposed a transversal implementation of the salutogenic approach in nursing curricula. It is also believed that the implementation of this approach at earlier ages (school) may help to encourage vocational choice in health-related studies, which we have observed to be associated with better academic achievement and higher SOC levels [32]. Salutogenic educative orientation expedites us to train students in order to reinforce their SOC and mainly, dynamize optimal HA against adversities and experiences that caring profession itself will make them live, in addition to improving their academic performance [59]. In this way, we will better prepare future healthcare professionals to be able to adapt to frequently hostile work environments and their internal threats, with a resilient capacity that will enable them to emerge strengthened from these experiences. This approach would reinforce their self-efficacy and self-esteem [17] and all of these would entail an indirect benefit for the health system itself and society [29].

### Study Limitations

One of the weaknesses of the present study is the individual recollection of HA data, but the strict bases of building up HA maps point at the recommendation of releasing it throughout participatory processes. Nor should be forgotten the importance of devolution of the results from mapping actions to the population in order to provoke reflection and raise awareness about their health potential. This aspect was not developed in this study due to the exploratory design. Another limitation is related to the cross-sectional study design, which hampers the ability to make causal inferences. Although these types of measures help enrich the perception of reality, they may, however, induce bias; while considering that a bias exists regarding the homogeneity of the sample in favor of female participants. On this, it is also convenient to highlight that one of the strengths of the present study is the use of mixed-methods combining the qualitative HA identification with another quantitative phase focused on exploring the relations between all HA categories reported with Antonovsky’s sense of coherence. This groundbreaking approach could allow obtaining new information of great interest in the fulfillment of the objectives of global salutogenic strategies.

## 5. Conclusions

The HA map articulated by CNA students was built and described. An implement that shall provide future opportunities for health and well-being among participants through empowering students in order to access and mobilize their resources and increasing their control over their health and its wider determinants. The relationships between primary HA, high SOC levels, a strong vocation for healthcare in concert with a heightened academic performance for this certification in public VET centers (Comunitat Valenciana, Spain) were confirmed. These consistent connections between the care–relation factor and vocational factor with specific HA reported, substantiate the salutogenic and asset-based approach. Ultimately, a salutogenic educative strategy root in: strengthen CNA student’s SOC, dynamize their HA map, reinforce their *sense of calling* which delves into the *zest for healthcare work,* consequently enable them to buffer against work-related caregiving stress and seems to be the proper to thrive in their profession.

## Figures and Tables

**Table 1:** Characteristics of the studied population.

Characteristics		N	%
Age group	Under 30	577	62.65
	30–45	219	23.78
	Over 45	125	13.57
Gender	Male	150	18.46
	Female	771	81.54
Geographic context	Rural areas	66	7.17
	Urban areas	520	56.46
	Large cities	335	36.37
Familiar income	Low	283	30.68
	Medium/Low	261	28.36
	Medium	297	32.19
	Medium/High	64	6.99
	High	16	1.78
Employment situation	Employed	222	24.11
	Unemployed	699	75.89
Career choice motivation	Vocational motivation	444	48.21
	Impossibility of access to other studies	21	2.28
	Seeking better work	316	34.31
	No motivation	25	2.71
	Other	115	12.49
Academic performance	Fail	25	2.71
	Pass	119	12.92
	Good	242	26.27
	Remarkable	303	32.89
	Outstanding	179	19.43
	With distinction	53	5.75

**Table 2:** Quantitative description of health assets (HA) identified by CNA students.

HA Category	HA Subcategory	N	%
Intrapersonal HA	Selfcare	14	1.52
	‘Caring for others’	82	8.91
	Others	825	89.57
Interpersonal HA	Couple	126	13.68
	Friends	59	6.41
	Ascendant relatives	165	17.91
	Descendant relatives	113	12.27
	Extended family	274	29.78
	Family + Friends	143	15.52
	Others	41	4.45
Extrapersonal HA I (Groups/Institutions)	Volunteering	47	5.10
	Educative institutions	33	3.58
	Sporting institutions	142	15.42
	Recreational/Leisure institutions	95	10.31
	Religious institutions	14	1.52
	Musical/artistic institutions	63	6.84
	Social group	396	42.99
	No assets identified	131	14.22
Extrapersonal HA II (Indoor/Outdoor spaces)	Mountain (natural space)	125	13.57
	Beach (natural space)	131	14.22
	Both mountain and beach	77	8.36
	Urban spaces	59	6.41
	Sporting spaces	63	6.84
	Recreational/leisure spaces	239	25.95
	Spiritual/religious spaces	6	0.65
	Educative spaces	15	1.63
	Cultural spaces	14	1.52
	Home	106	11.51
	Working place	10	1.08
	No assets identified	76	8.25

**Table 3:** HA identified by CNA students according to their gender, age group and employment situation.

HA Category	HA Subcategory	Male	Female		Under 30	30–45	Over 45		Employed	Unemployed	
		%	%	*p*	%	%	%	*p*	%	%	*p*
Intrapersonal HA	Selfcare	2.35	1.33	0.302	1.04	2.28	2.4	0.629	1.8	1.43	0.559
	‘Caring for others’	6.47	9.45		9.01	8.22	9.6		7.21	9.44	
Interpersonal HA	Couple	14.11	13.58	0.001	12.82	16.44	12.8	0.000	11.71	14.31	0.029
	Friends	12.94	4.93		6.76	6.39	4.8		6.31	6.44	
	Ascendant relatives	23.53	16.64		21.66	15.07	5.6		12.16	19.74	
	Descendant relatives	7.65	13.31		3.29	22.37	36		17.12	10.73	
	Extended family	24.71	30.89		32.93	22.83	27.2		32.43	28.89	
Extrapersonal HA I Group/institutions	Sporting institutions	22.94	13.71	0.021	14.9	18.72	12	0.003	15.31	15.45	0.030
	Recreational/Leisure inst.	11.76	9.99		10.05	9.59	12.8		8.55	10.87	
	Social group	32.94	45.27		46.45	34.7	41.6		39.18	44.21	
	Volunteering	5.29	5.06		3.29	7.82	8.8		7.66	4.29	
Extrapersonal HA II Indoor/Outdoor spaces	Mountain spaces	15.29	13.18	0.075	10.05	18.72	20.8	0.000	12.61	13.88	0.012
	Beach spaces	11.76	14.78		15.42	14.15	8.8		15.31	13.87	
	Recreational/Leisure space	25.29	26.09		30.5	19.18	16.8		22.07	27.18	
	Home	11.76	11.45		12.48	8.67	12		8.56	12.44	

**Table 4:** SOC scores, according to intrapersonal HA identified by CNA students.

HA	SOC (Global)			Manageability			Comprehensibility			Meaningfulness		
Subcategory	Mean	SD	*p*	Mean	SD	*p*	Mean	SD	*p*	Mean	SD	*p*
Selfcare	56.07	7.79	0.95	16.36	4.38	0.48	17.71	4.41	0.54	22	2.93	0.08
‘Caring for others’	56.89	12.68		15.82	4.75		19.56	5.61		21.51	4.61	
Others	56.34	12.26		16.52	4.51		19.27	5.66		20.55	4.49	

**Table 5:** SOC scores, according to interpersonal HA identified by CNA students.

HA	SOC (Global)			Manageability			Comprehensibility			Meaningfulness		
Subcategory	Mean	SD	*p*	Mean	SD	*p*	Mean	SD	*p*	Mean	SD	*p*
Couple	55.47	13.75	0.003	15.91	5	0.004	18.80	6.10	0.035	20.75	4.55	0.001
Friends	54.39	12.35		16.02	4.83		19.05	5.27		19.32	5	
Ascendant relatives	54.96	10.99		16.02	4.43		19.15	5.10		19.79	4.17	
Descendant relatives	60.76	11.83		17.79	4.36		21.04	5.76		21.94	4.24	
Extended family	56.24	12.08		16.43	4.40		19.14	5.45		20.68	4.51	
Family + Friends	56.65	12.32		16.71	4.43		18.77	6.12		21.17	4.22	
Others	55.68	11.73		16.02	4.17		19.39	5.43		20.27	5.21	

**Table 6:** Post hoc analysis. Differences in SOC scores according to interpersonal HA identified by CNA, students, considering as reference the scores obtained by those who consider their descendent relatives as HA.

HA	SOC (Global)		Manageability		Comprehensibility		Meaningfulness	
Subcategory	* Mean Differences	*p*	* Mean Differences	*p*	* Mean Differences	p	* Mean Differences	*p*
Couple	5.29	0.002	1.88	0.001	2.24	0.003	1.19	0.055
Friends	6.37	0.002	1.76	0.013	1.99	0.034	2.62	0.001
Ascendant relatives	5.8	0.000	1.77	0.000	1.89	0.004	2.15	0.000
Extended family	2.15	0.001	1.36	0.002	1.9	0.002	1.26	0.015
Family + Friends	4.11	0.008	1.08	0.029	2.27	0.002	0.77	0.141
Others	5.08	0.014	1.77	0.004	1.35	0.097	1.67	0.084

* Mean differences are the differences between the scores obtained by CNA students who identified descendent relatives (reference group) as HA and the scores obtained by those who identified the HA shown in each row.

**Table 7:** SOC scores, according to extrapersonal HA identified by CNA students.

HA	SOC (Global)			Manageability			Comprehensibility			Meaningfulness		
Category/Subcategory	Mean	SD	*p*	Mean	SD	*p*	Mean	SD	*p*	Mean	SD	*p*
Groups/institutions (HA I)												
Volunteering	57.96	13.77	0.038	16.02	4.52	0.204	20.77	6.05	0.002	21.17	4.49	0.138
Educative institutions	57.73	13.08		15.70	5.47		19.88	6.22		22.15	4.44	
Sporting institutions	57.58	12.20		16.80	4.62		19.93	5.40		20.85	4.54	
Recreational/leisure institutions	57.35	11.85		16.94	4.47		19.64	5.27		20.77	4.08	
Religious institutions	49.93	12.44		14.71	4.39		14.36	6.12		20.86	4.27	
Musical/artistic institution	53.87	13.05		15.79	4.94		18.13	5.95		19.95	4.52	
Social group	56.77	11.87		16.70	4.32		19.32	5.60		20.75	4.48	
No assets identified	54.21	12.10		15.84	4.59		18.56	5.47		19.81	4.68	
Indoor/Outdoor Spaces (HA II)			0.037			0.093			0.075			0.099
Mountain (natural space)	58.19	12.36		16.94	4.40		20.12	5.82		21.13	4.29	
Beach (natural space)	55.75	11.05		16.04	4.57		18.81	4.82		20.90	4.23	
Both mountain and beach	58.88	11.51		17.09	4.48		20.13	5.93		21.66	3.94	
Urban/rural spaces	56.36	14.77		17.02	5.01		18.76	6.34		20.58	5.56	
Sporting spaces	60.37	12.98		18.02	4.73		21.06	5.87		21.29	4.73	
Recreational/leisure spaces	54.54	11.63		15.84	4.25		18.69	5.52		20.02	4.64	
Spiritual/religious spaces	60.83	15.20		17.33	6.28		20.50	7.18		23	2.68	
Educative spaces	55.27	12.96		16	5.37		17.33	6.14		21.93	3.95	
Cultural spaces	54.50	13.51		15.79	4.89		18.07	7.24		20.64	3.05	
Home	55.92	11.90		16.27	4.35		19.41	5.09		20.25	4.39	
Working place	59.70	14.21		17.70	3.33		20.60	6.45		21.40	5.38	
No assets identified	54.87	12.50		16.14	4.78		18.72	5.78		20	4.38	

## References

[1] Bauer G.F., Roy M., Bakibinga P., Contu P., Downe S., Eriksson M., Espnes G.A., Jensen B.B., Juvinya Canal D., Lindström B. (2019). Future Directions for the Concept of Salutogenesis: A Position Article. Health Promot. Int..

[2] Van Bortel T., Wickramasinghe N.D., Morgan A., Martin S. (2019). Health Assets in a Global Context: A Systematic Review of the Literature. BMJ Open.

[3] Kretzmann J.P., McKnight J.L. (1993). Building Communities from the Inside Out: A Path Toward Finding and Mobilizing a Community’s Assets.

[4] Morgan A., Ziglio E. (2007). Revitalising the Evidence Base for Public Health: An Assets Model. Promot. Educ..

[5] Pérez-Wilson P., Hernán M., Morgan A.R., Mena A. (2015). Health Assets for Adolescents: Opinions from a Neighbourhood in Spain. Health Promot. Int..

[6] Rivera de los Santos F., Ramos Valverde P., Moreno Rodríguez C., Hernán García M. (2011). Análisis Del Modelo Salutogénico En España: Aplicación En Salud Pública E Implicaciones Para El Modelo De Activos En Salud. Rev. Española Salud Pública.

[7] Mittelmark M.B., Bauer G.F., Mittelmark M.B. (2017). The meanings of salutogenesis. The Handbook of Salutogenesis.

[8] Idan O., Eriksson M., Al-Yagon M., Mittelmark M.B. (2017). The salutogenic model. The Handbook of Salutogenesis..

[9] Antonovsky A. (1987). Unraveling the Mystery of Health: How People Manage Stress and Stay Well.

[10] Antonovsky A. (1996). The Salutogenic Model as a Theory to Guide Health Promotion. Health Promot. Int..

[11] Hernan M., Morgan A., Mena A.L. (2010). Formación En Salutogénesis Y Activos Para La Salud.

[12] Lindström B., Eriksson M. (2011). From health education to healthy learning: Implementing salutogenesis in educational science. Scand. J. Public Health.

[13] Cofiño R., Aviñó D., Benedé C.B., Botello B., Cubillo J., Morgan A., Paredes-Carbonell J.J., Hernán M. (2016). Promoción De La Salud Basada En Activos: ¿cómo Trabajar Con Esta Perspectiva En Intervenciones Locales?. Gac. Sanit..

[14] Cassetti V., Powell K., Barnes A., Sanders T. (2019). A Systematic Scoping Review of Asset-Based Approaches to Promote Health in Communities: Development of a Framework. Glob. Health Promot..

[15] Lazarus R.S., Folkman S. (1984). Stress, Appraisal, and Coping.

[16] Lazarus R.S. (2006). Emotions and Interpersonal Relationships: Toward a Person-Centered Conceptualization of Emotions and Coping. J. Personal..

[17] Lindström B., Eriksson M. (2006). Contextualizing Salutogenesis and Antonovsky in Public Health Development. Health Promot. Int..

[18] Mittelmark M., Bull T., Bouwman L.I., Mittelmark M.B. (2017). Emerging Ideas Relevant to the Salutogenic Model of Health. The Handbook of Salutogenesis.

[19] Morgan A. (2014). Revisiting the Asset Model: A Clarification of Ideas and Terms. Glob. Health Promot..

[20] Todahl J.L., Walters E., Bharwdi D., Dube S.R. (2014). Trauma Healing: A Mixed Methods Study of Personal and Community-Based Healing. J. Aggress. Maltreatment Trauma.

[21] Sakallaris B.R., Macallister L., Voss M., Smith K., Jonas W.B. (2015). Optimal Healing Environments. Glob. Adv. Health Med..

[22] Parkin S. (2015). Salutogenesis: Contextualising Place and Space in the Policies and Politics of Recovery from Drug Dependence (UK). Int. J. Drug Policy.

[23] Boscherini G. (2017). A Sense of Coherence: Supporting the Healing Process. Archit. Des..

[24] Dube S.R., Rishi S. (2017). Utilizing the Salutogenic Paradigm to Investigate Well-being among Adult Survivors of Childhood Sexual Abuse and Other Adversities. Child Abus. Negl..

[25] Rudman A., Gustavsson J.P. (2012). Burnout during Nursing Education Predicts Lower Occupational Preparedness and Future Clinical Performance: A Longitudinal Study. Int. J. Nurs. Stud..

[26] Pulido-Martos M., Augusto-Landa J.M., Lopez-Zafra E. (2012). Sources of Stress in Nursing Students: A Systematic Review of Quantitative Studies. Int. Nurs. Rev..

[27] Chana N., Kennedy P., Chessell Z.J. (2015). Nursing Staffs’ Emotional Well-being and Caring Behaviours. J. Clin. Nurs..

[28] Lee Y.E., Kim E., Park S.Y. (2017). Effect of Self-Esteem, Emotional Intelligence and Psychological Well-being on Resilience in Nursing Students. Child Health Nurs. Res..

[29] Bakibinga P., Vinje H.F., Mittelmark M.B. (2012). Self-Tuning for Job Engagement: Ugandan Nurses’ Self-Care Strategies in Coping with Work Stress. Int. J. Ment. Health Promot..

[30] Vinje H.F., Ausland L.H., Langeland E., Mittelmark M.B. (2016). The Application of Salutogenesis in the Training of Health Professionals. The Handbook of Salutogenesis.

[31] Jenny G.J., Bauer G.F., Vinje H.F., Vogt K., Torp S., Mittelmark M.B. (2016). The application of salutogenesis to work. The Handbook of Salutogenesis.

[32] Colomer-Pérez N., Paredes-Carbonell J.J., Sarabia-Cobo C., Gea-Caballero V. (2019). Sense of Coherence, Academic Performance and Professional Vocation in Certified Nursing Assistant Students. Nurse Educ. Today.

[33] Colomer-Perez N., Paredes-Carbonell J.J., Sarabia-Cobo C.M., Gea-Caballero V. Self-Care Agency and Sense of Coherence Implications for a Salutogenic Model of Care in Certified Nursing Assistant Students. Health Educ. Behav..

[34] Antonovsky A. (1993). The Structure and Properties of the Sense of Coherence Scale. Soc. Sci. Med..

[35] Lizarbe-Chocarro M., Guillén-Grima F., Aguinaga-Ontoso I., Canga Armayor N. (2016). Validación Del Cuestionario De Orientación a La Vida (OLQ-13) De Antonovsky En Una Muestra De Estudiantes Universitarios En Navarra. An. Sist. Sanit. Navar..

[36] Eriksson M., Lindström B. (2005). Validity of Antonovsky’s Sense of Coherence Scale: A Systematic Review. J. Epidemiol. Community Health.

[37] Morgan A., Ziglio E., Davies M. (2010). Health Assets in a Global Context.

[38] Blickem C., Dawson S., Kirk S., Vassilev I., Mathieson A., Harrison R., Bower P., Lamb J. (2018). What is Asset-Based Community Development and how might it Improve the Health of People with Long-Term Conditions? A Realist Synthesis. SAGE Open.

[39] McKnight J.L., Russell C. (2018). The Four Essentian Elements of an Asset-Based Community Developement Process.

[40] Paredes-Carbonell J.J., Agulló-Cantos J.M., Vera-Remartínez E.J., Hernán-García M. (2013). Sentido De Coherencia Y Activos Para La Salud En Jóvenes Internos En Centros De Menores. Rev. Española Sanid. Penit..

[41] Agulló-Cantos J.M., García-Alandete J., Paredes-Carbonell J.J. (2019). Activos Para La Salud En Cuidadores Familiares De Enfermos De Alzheimer: Desarrollo De Un Mapa De Activos Para La Salud. Glob. Health Promot..

[42] Alpuente A.C., Cintas F.A., Foà C., Cosentino C. (2018). Mapping Caregivers’ Health Assets. A Self-Care Project using Salutogenesis and Mindfulness. Acta Bio Med. Atenei Parm..

[43] Natvig G.K., Albrektsen G., Qvarnstrøm U. (2003). Associations between Psychosocial Factors and Happiness among School Adolescents. Int. J. Nurs. Pract..

[44] Moksnes U.K., Rannestad T., Byrne D.G., Espnes G.A. (2011). The Association between Stress, Sense of Coherence and Subjective Health Complaints in Adolescents: Sense of Coherence as a Potential Moderator. Stress Health.

[45] Torsheim T., Aaroe L.E., Wold B. (2001). Sense of Coherence and School-Related Stress as Predictors of Subjective Health Complaints in Early Adolescence: Interactive, Indirect or Direct Relationships?. Soc. Sci. Med..

[46] Kristensson P., Öhlund L.S. (2005). Swedish Upper Secondary School Pupils’ Sense of Coherence, Coping Resources and Aggressiveness in Relation to Educational Track and Performance. Scand. J. Caring Sci..

[47] Vinje H.F. (2007). Thriving Despite Adversity: Job Engagement and Self-Care among Community Nurses. Ph.D. Thesis.

[48] Oliva Delgado A., Jiménez Morago J.M., Parra Jiménez A., Sánchez-Queija I. (2008). Acontecimientos Vitales Estresantes, Resiliencia Y Ajuste Adolescente. Rev. Psicopatol. Psicol. Clínica.

[49] Reina M.D.C., Oliva-Delgado A., Parra-Jiménez Á. (2017). Percepciones De Autoevaluación: Autoestima, Autoeficacia Y Satisfacción Vital En La Adolescencia. Psychol. Soc. Educ..

[50] Gonzalez M.P., Rey Yedra L. (2006). La Escuela Y Los Amigos: Factores Que Pueden Proteger a Los Adolescentes Del Uso De Sustancias Adictivas. Ensen. Investig. Psicol..

[51] Moyano Díaz E., Ramos Alvarado N. (2007). Bienestar Subjetivo: Midiendo Satisfacción Vital, Felicidad Y Salud En Población Chilena De La Región Maule. Universum Talca.

[52] Vera-Remartínez E.J., Paredes-Carbonell J.J., Aviñó Juan-Ulpiano D., Jiménez-Pérez M., Araujo Pérez R., Agulló-Cantos J.M., Mora Notario A. (2017). Sentido De Coherencia Y Mapa De Activos Para La Salud En Jóvenes Presos De La Comunidad Valenciana En España. Glob. Health Promot..

[53] García-Moya I., Rivera F., Moreno C., López A. (2013). Calidad De La Relación Entre Los Progenitores Y Sentido De Coherencia En Sus Hijos Adolescentes. El Efecto De Mediación De La Satisfacción Familiar. An. Psicol..

[54] Malagón Aguilera M.C. (2016). El Sentido De La Coherencia Y El Compromiso Laboral De Las Enfermeras En El Ámbito Sociosanitario De Girona. Ph.D. Thesis.

[55] Restrepo H.E., Málaga H. (2001). Promoción De La Salud: Cómo Construir Vida Saludable.

[56] Paakkari L., Torppa M., Välimaa R., Villberg J., Ojala K., Tynjälä J. (2019). Health Asset Profiles and Health Indicators among 13- and 15-Year-Old Adolescents. Int. J. Public Health.

[57] Kim H., Ryu S., Jang K. (2019). Effect of Structured Pre-Simulation Preparation and Briefing on Student’s Self-Confidence, Clinical Judgment, and Clinical Decision-Making in Simulation. Contemp. Nurse.

[58] Mayer C., Boness C. (2011). Interventions to Promoting Sense of Coherence and Transcultural Competences in Educational Contexts. Int. Rev. Psychiatry.

[59] Caan W., Cassidy J., Coverdale G., Ha M., Nicholson W., Rao M. (2014). The Value of using Schools as Community Assets for Health. Public Health.

